# Effect of surface-modified intraocular lenses on long-term postoperative inhibition of posterior capsule opacification^☆^

**DOI:** 10.1016/j.heliyon.2024.e33006

**Published:** 2024-06-14

**Authors:** Mayumi Nagata, Hiroyuki Matsushima, Tadashi Senoo

**Affiliations:** Department of Ophthalmology, Dokkyo Medical University, 880 Kitakobayashi, Mibu City, Tochigi, 321-0293, Japan

**Keywords:** Posterior capsule opacification, Intraocular lens, Neodymium–yttrium–aluminum–garnet capsulotomy, Surface modification, Scattered light intensity

## Abstract

We compared the posterior capsule opacification incidences at 5 years postoperatively and the neodymium–yttrium–aluminum–garnet capsulotomy rates at 10 years postoperatively for two types of intraocular lenses with different optical properties and shapes. This randomized, controlled, prospective, single-blinded study with intra-individual comparisons was conducted between July 21, 2009, and August 31, 2011, at the Dokkyo Medical University Hospital, Tochigi, Japan. Thirty patients (60 eyes) underwent bilateral cataract surgery and received a XY1 intraocular lens in one eye and a FY-60AD intraocular lens in the other. Both intraocular lenses are acrylic and manufactured by HOYA. The XY1 lens is a single-piece, tinted intraocular lens featuring an ultraviolet/ozone treatment on the posterior surface of the lens optic, aimed at enhancing posterior capsule adhesion to prevent posterior capsule opacification. Conversely, the FY-60AD is a tinted intraocular lens with modified polymethylmethacrylate C-loops and no ultraviolet/ozone treatment of the optic. Scheimpflug images were taken using EAS-1000 (NIDEK Co., Ltd., Aichi, Japan), and the scattered light intensity (computer compatible tape) on the posterior surface of the intraocular lens was calculated and evaluated as the posterior capsule opacification. The scattered light values of the XY1 and FY-60AD groups were 6.50 ± 5.69 and 11.64 ± 5.30 computer compatible tape, respectively, at 5 years postoperatively. The cumulative survival incidence after neodymium–yttrium–aluminum–garnet laser capsulotomy was 74.8 % in the XY1 group and 13.8 % in the FY-60AD group at 10 years postoperatively. The surface-modified intraocular lens XY1 reduced the incidence of posterior capsule opacification even 10 years after surgery. Surface modification to increase the adhesion between the intraocular lens and the capsule effectively prevents posterior capsule opacification.

## 1 Introduction

Advancements in surgical equipment and intraocular lenses (IOLs) for cataract surgery have made it possible to achieve good visual function in the early postoperative period. The age range for intraocular lens implantation has expanded and the effects on visual function in children and cognitive function in older adults have also been observed [1,2]. However, posterior capsule opacification (PCO) is an inevitable postoperative complication after cataract surgery, which reduces postoperative visual function [3,4]. In the early postoperative period, the wound healing response produces cytokines, such as transforming growth factor-β and basic fibroblast growth factor, which transform the lens epithelial cells (LECs) into fibroblast-like cells, resulting in fibrous opacity (epithelial-mesenchymal transition). In the late postoperative period, 1 year or more, LECs that had proliferated in the IOL area had proliferated around the posterior capsule, causing PCO [5,6]. Currently, conventional IOLs possess modifications, such as a square edge to prevent LEC proliferation [7,8]. It is impossible to completely prevent PCO by using a square edge, as it progresses gradually over a long period following surgery, requiring neodymium-yttrium-aluminum-garnet (Nd:YAG) laser capsulotomy in approximately 20 % of cases at 5 years postoperatively.[9]^.^

The Nd:YAG laser capsulotomy is effective for PCO; however, it can lead to complications such as anterior chamber inflammation, increased intraocular pressure, macular edema, and retinal detachment [10,11]. In addition, Nd:YAG laser capsulotomy is challenging to perform in children and older patients who cannot remain at rest. Therefore, PCO inhibition is important for the long-term maintenance of postoperative visual function, which requires methods other than square edges.

Vivinex (XY1, HOYA Surgical Optics, Chromos, Singapore) is a hydrophobic acrylic IOL that has square edges and prevents PCO by surface modification of the posterior surface of the IOL optic to improve adhesion between the IOL optic and posterior capsules, and eliminates space for LEC proliferation [12]. The XY1 IOL is expected to prevent long-term PCO, and its efficacy in inhibiting PCO at 3–5 years postoperatively has been reported [[13], [14], [15]]; however, there are no reports on PCO occurrences in the longer postoperative courses of a XY1.

This study aimed to evaluate the long-term PCO prevention effect of XY1. However, because the XY1 is constructed of a newly developed acrylic material by HOYA Surgical Optics, IOLs made of the same material without surface modification cannot be used clinically as a comparison. Therefore, the FY-60AD, which is constructed of a conventional acrylic material made by HOYA Surgical Optics, was selected for comparison and contrast. This study examined the incidence of PCO at 5 years postoperatively and the incidence of Nd:YAG laser capsulotomy at 10 years postoperatively in the same patients with XY1 in one eye and FY-60AD, an IOL without surface modification, in the other eye.

## 2 Materials and methods

This observational study included patients who participated in a randomized clinical trial approved by the Institutional Review Board of Dokkyo Medical University (approval no. R-69-5J). This study was conducted in accordance with the tenets of the Declaration of Helsinki. Written informed consent was obtained from all patients for participation in this study and for publication of their images.

### 2.1 Patients

Thirty patients (60 eyes) participated in this randomized, controlled, prospective, single-blind study conducted between July 21, 2009, and August 31, 2011. The patients underwent cataract surgery between September 2009 and May 2010.

Patients with serious systemic diseases such as diabetes and uncontrolled hypertension, or ocular diseases such as glaucoma, corneal opacity, diabetic retinopathy, macular disease, and uveitis were excluded. An envelope containing a piece of paper with randomly assigned IOLs (XY1 or FY-60AD) prepared by the investigator was opened at the time of surgery, and the assigned IOL was inserted into the first eye. Another intraocular lens was implanted in the second eye. All patients underwent cataract surgery in one eye, followed by surgery in the other eye the next day.

### 2.2 Surgical technique and postoperative treatment

Surgery was performed by a skilled surgeon. After administering eye drops or sub-Tenon's anesthesia, a 2.4 mm corneoscleral incision was created. A complete 5 mm-diameter continuous curvilinear capsulorhexis was created after filling the anterior chamber with Viscoat^Ⓡ^ (Alcon Laboratories, Inc., Fort Worth, TX, USA) and HEALON^Ⓡ^ (Johnson & Johnson Vision, Irvine, CA, USA) ophthalmic viscoelastic devices. After hydrodissection, phacoemulsification and cortical aspiration were performed. After filling the lens capsule with HEALON^Ⓡ^ (Johnson & Johnson Vision) and inserting the IOL into the capsule, the ophthalmic viscoelastic devices in the anterior chamber was removed by irrigation/aspiration. No intra-operative complications were observed in any case.

Postoperative eye drops consisted of 1.5 % levofloxacin hydrate (Santen Pharmaceutical Co., Ltd., Osaka, Japan) and 0.1 % betamethasone sodium phosphate (Shionogi & Co., Ltd., Osaka, Japan), a steroid, five times per day for 2 weeks, and a non-steroidal anti-inflammatory drug (NSAIDs), 0.1 % bromfenac sodium hydrate (Senjyu Pharmaceutical Co., Ltd., Osaka, Japan) was continued twice a day for 3 months. No patient showed severe intraocular inflammation on postoperative examination.

Although it has been reported that steroid suppresses PCO development and that the combination of steroids with NSAIDs lacks PCO suppression effect [16], in this study, patients were administered NSAIDs in combination with steroids to suppress postoperative cystoid macular edema [17].

### 2.3 Specifications of the implanted IOLs

**Fig. 1:**
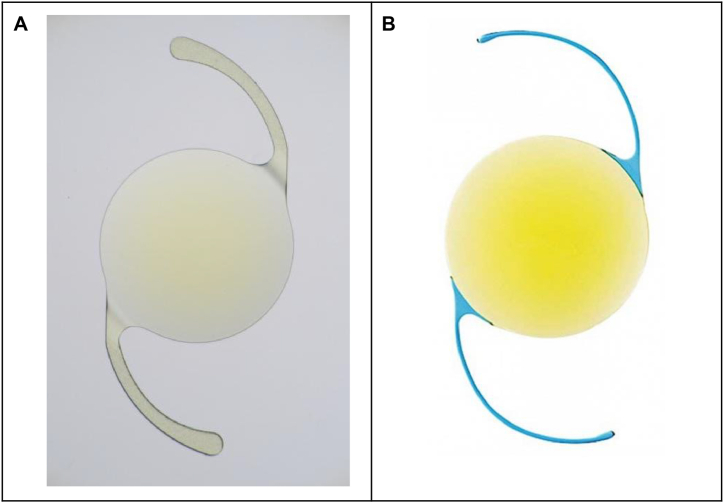
Two types of intraocular lens images validated in this study A. XY1 B. FY-60AD.

Fig. 1 shows the images of the two IOLs (XY1 and FY-60AD) used in this study. XY1 is a single-piece tinted IOL made of hydrophobic acrylic (Fig. 1A). The optic is 6.0 mm in diameter and 13 mm in overall length, with C-Loop haptics of the same material and no optic-haptic junction. As a major optic characteristic, the XY1 uses ultraviolet (UV)/ozone (O3) to modify the posterior surface of the IOL optic to prevent PCO and improve posterior capsule adhesion. The UV/O3 treatment improves wettability and adhesion because active species such as activated oxygen and ozone, generated by the UV irradiation and sterilization improve the surface properties and produce hydroxol and carboxyl functional groups [18].

The FY-60AD is a tinted IOL made of hydrophobic acrylic (Fig. 1B). The optic is 6.0 mm in diameter and 12.5 mm in overall length, with modified polymethylmethacrylate C-loops and no optic-haptic junction. The FY-60AD is a preloaded IOL that has been discontinued, but the PY-60AD (HOYA Surgical Optics) is currently in clinical use. It has the exact specifications as the FY-60AD, offering a comparable IOL option.

### 2.4 PCO analysis

Scheimpflug images were taken at two meridian positions (0° and 90°) using EAS-1000 (NIDEK Co., Ltd., Aichi, Japan) every other year for 5 years postoperatively.

In order to analyze the progression of PCO to the pupillary area**,** which can lower the patient's visual acuity, mydriasis was induced using ophthalmic drops containing a combination of tropicamide and phenylephrine (Mydrin®-P, Santen Pharmaceutical, Osaka, Japan). Next, using the densitometry analysis mode in the EAS-1000, the scattered light intensity in a 3.0 mm diameter × 0.25 mm depth region of the posterior chamber (Computer Compatible Tape [CCT]) was calculated, and the average of two measurements was used as the PCO value [19]. The PCO values were then compared between the two types of IOLs.

Patients who underwent Nd:YAG laser capsulotomy during the 5-year follow-up period were excluded from PCO analysis. The EAS-1000 broke in June 2015 and could not be repaired; therefore, subsequent analyses were not possible.

### 2.5 Observation of early PCO by anterior segment photography

For 10 years postoperatively, retroillumination images were taken using a digital photo slit lamp RS-1000 (Right Group, Tokyo, Japan) to observe the PCO state.

Since it has been reported that early PCO tends to originate from the optic-haptic junction area, where square edged shape effects are less likely to appear [20], we paid particular attention to PCO occurrence in the optic-haptic junction area.

### 2.6 Cumulative survival curves of Nd:YAG laser capsulotomy

The Nd:YAG laser capsulotomy was performed at the physician's discretion in the cases that PCO was detected by slit-lamp examination with a decrease in visual acuity from the best postoperative acuity [14], or the patient complained of decreased visual function and no cause other than PCO was identified. The timing of the Nd:YAG laser capsulotomy was determined from the medical records.

Cumulative survival rates for Nd:YAG laser capsulotomy at 10 years postoperatively were determined for the two different lens groups using Kaplan–Meier curves. Kaplan–Meier survival distribution plots were generated to display the time to Nd:YAG laser capsulotomy events with adjustment for censoring. Cases of patients with self-interruption during hospital visits and deaths were included in the censored data.

### 2.7 Sample size and statistical analyses

The sample size was calculated using EZR software (version 1.61; Saitama City, Saitama Prefecture, Japan) [21] and the same software was used for statistical analyses. The patient number was selected to detect a difference in the PCO percentage of 40 % between groups after 10 years based on a previous study [8]. The α error was set to 0.05; therefore, at least 20 patients were required for a power of 0.8. Thirty patients were included in this study, accounting for a dropout rate of 50 %. Welch's *t*-test was used for the statistical analysis of PCO and the log-rank test was used to analyze the cumulative survival curves of Nd:YAG laser capsulotomy. Statistical significance was set at *p* < 0.05.

## 3 Results

### 3.1 Patient demographic data

**Fig. 2:**
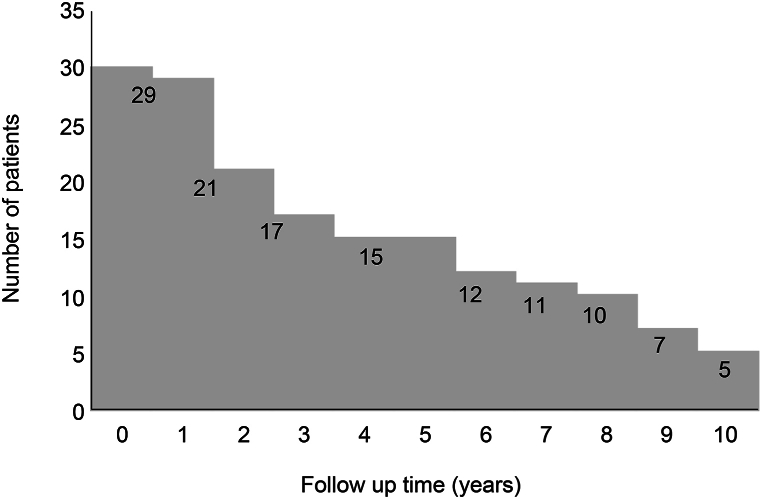
Number of patients during the follow-up period.

Of the 30 patients (mean age: 71.4 ± 8.65 years, 20 women), 16 had a XY1-implanted in the right eye and a FY-60AD implanted in the left eye, whereas 14 had a FY-60AD in the right eye and a XY1-implanted in the left eye (Table 1). Fig. 2 presents the number of patients during the follow-up periods. The number of patients who interrupted their hospital visits during the 10-year period was 15, and 5 patients were available for examination at 10 years postoperatively. The causes of hospital visit interruptions were dementia progression in 1 patient, 3 patient deaths, and 21 with unknown reasons for interruptions.

**Table 1:** Patient demographic data.

Total	Mean age: 71.4 ± 8.65 years	30 cases
Males	Mean age: 68.6 ± 10.2 years	10 cases
Females	Mean age: 72.9 ± 7.68 years	20 cases
Right: XY1 Left: FY-60AD	Mean age: 73.4 ± 7.86 years	16 cases
Right: FY-60AD Left: XY1	Mean age: 69.2 ± 9.25 years	14 cases

### 3.2 PCO analysis

**Fig. 3:**
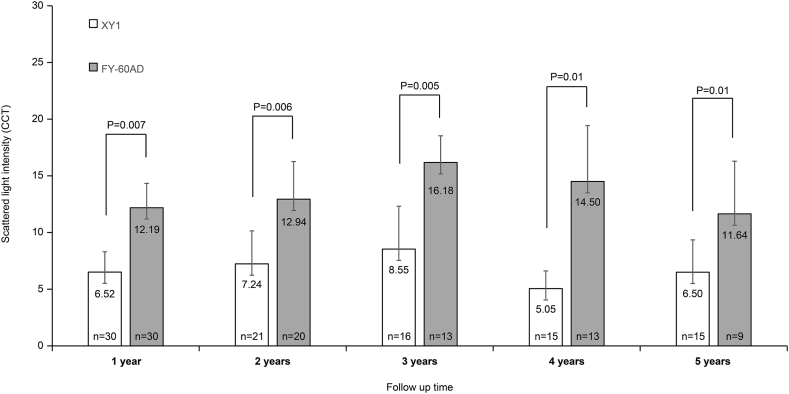
Welch's *t*-test of posterior capsule opacification analysis. The scattered light values in the XY1 group were significantly lower than that in the FY-60AD group during all observation periods. Data presented as mean ± SD. CCT: computer compatible tape.

The scattered light values (CCT) in the XY1 group were significantly lower than those in the FY-60AD group at 1 year (6.52 ± 3.58 vs. 12.19 ± 4.30, *p* = 0.007), 2 years (7.24 ± 5.82 vs. 12.94 ± 6.65, *p* = 0.006), 3 years (8.55 ± 7.54 vs. 16.18 ± 4.70, *p* = 0.005), 4 years (5.05 ± 3.13 vs. 14.50 ± 6.86, *p* = 0.01), and 5 years (6.50 ± 5.69 vs. 11.64 ± 5.30, *p* = 0.01) (Fig. 3).

### 3.3 Observation of early PCO by anterior segment photograph

**Fig. 4:**
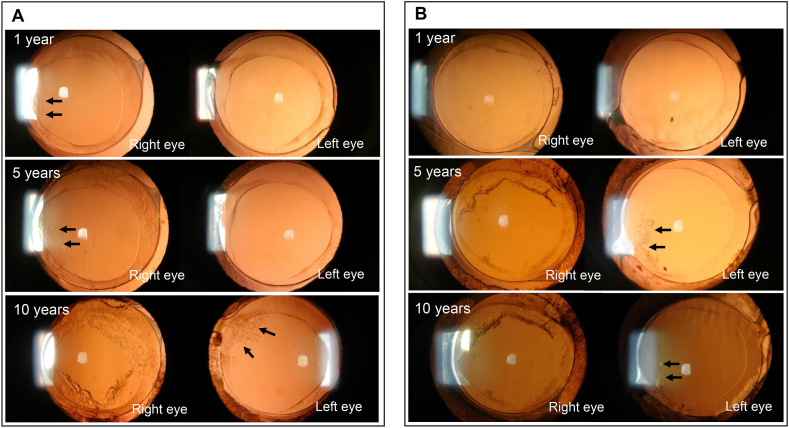
Observation of PCO by anterior segment photograph for 2 representative cases. A. Case 1: FY-60AD-implanted in the right eye and XY-implanted in the left eye. B. Case 2: FY-60AD-implanted in the right eye and XY-implanted in the left eye. Black arrows: PCO development from optic-haptic junction; PCO: posterior capsule opacification.

Fig. 4 shows an anterior segment photograph of two representative cases (Fig. 4A and B) implanted with a FY-60AD in the right eye and a XY1 in the left eye at 1, 5, and 10 years postoperatively. In case B, where a XY1 was implanted in the left eye, the presence of PCO originating from the optic-haptic junction of the IOL was observed 5 years postoperatively (5 years, black arrows), but did not progress even at 10 years postoperatively (10 years, black arrows).

### 3.4 Cumulative survival curves of Nd:YAG laser capsulotomy

**Fig. 5:**
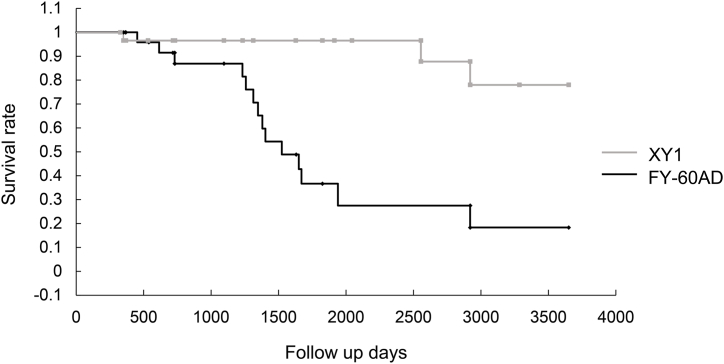
Cumulative survival curves of neodymium-yttrium-aluminum-garnet (Nd:YAG) laser capsulotomy. Kaplan–Meier survival plot of time to Nd:YAG laser capsulotomy of XY1 versus FY-60AD intraocular lenses.

The cumulative survival rates after Nd:YAG laser capsulotomy were 74.8 % in the XY1 group and 13.8 % in the FY-60AD group. Fig. 5 shows a Kaplan–Meier plot of the time to Nd:YAG laser capsulotomy in the XY1 group compared to the FY-60AD group.

## 4 Discussion

In this study, the XY1 and FY-60AD IOLs were randomly implanted in both eyes of the same patient. The PCO analysis using Scheimpflug images 5 years postoperatively indicated that the XY1 had significantly lower scattered light values than the FY-60AD.

PCO progresses from the optic-haptic junction site without a square edge shape [20]; however, observation of PCO development using anterior segment photographs revealed LEC proliferation from the optic-haptic junction (Fig. 4). The YAG was not performed in the XY1-implanted eye because the progression was slow and did not affect visual function. The PCO incidences were lower in the XY1-implanted eyes and the cumulative survival incidence of Nd:YAG laser capsulotomy 10 years postoperatively was higher for the XY1 than for the FY-60AD.

The PCO inhibition effect of the square edge has been previously reported [7,8], and employed in many IOLs. However, the inhibition effect is not constant because the sharpness of the square edges varies with the IOL type, and many IOLs have a low degree of sharpness [22]. In addition, regardless of edge design, the ratio of YAG laser capsulotomy increases over time postoperatively [23]. Therefore, the PCO inhibition effect of the square edge is limited, and other methods must be added.

Linnola et al. [24] proposed the sandwich theory to prevent PCO after cataract surgery. This theory suggests that a sandwich pattern comprising the IOL, LEC layer, and posterior capsule promotes adhesion after surgery and prevents PCO formation. A single LEC layer acts as an adhesive to promote adhesion between the IOL and posterior capsule rather than direct contact between the IOL and posterior capsule. In addition to LECs, proteins constituting the extracellular matrix, including fibronectin, vitronectin, laminin, and collagen IV play important roles in cell adhesion [24,25]. Based on this theory, a surface modification method was devised to prevent PCO using UV ozone treatment to improve adhesion between the intraocular lens and posterior capsule [12,26]. In UV ozone treatment, active species such as active oxygen and ozone generated by UV irradiation and sterilization improve surface properties to generate hydroxol and carboxyl functional groups, which have the effect of improving wettability and adhesion. Therefore, the increased wettability of the material surface results in a smaller contact angle and stronger adhesion [18].

We analyzed PCO occurrence in acrylic material IOLs, which have strong LEC adhesion [23], by inserting them into albino rabbits after UV ozone and argon plasma treatments, which are surface modification methods. The results showed that the UV ozone treatment inhibited PCO more than the argon plasma treatment and did not cause any damage to the acrylic material [12,26]. Eldred et al. [27] implanted a XY1 and AcrySof into the human capsular bag model using human donors and cultured cells, and reported that cell migration to the posterior capsule was slower in the XY1 than in AcrySof. In the present study, the surface modified XY1 had higher PCO inhibition at 10 years postoperatively compared to other IOL implanted in the same patient, demonstrating the effectiveness of surface modification over the long postoperative period.

Several clinical studies on PCO in a XY1 have been reported (Table 2). Leydolt et al. [13] conducted a randomized study implanting a AcrySof SN60WF (Alcon Laboratories) in one eye and XY1 in the other eye of the same patient. They quantified the incidence of PCO at 3 years postoperatively using the automated image analysis software (AQUA–Automated Quantification of After-Cataract; Institute for Computer Graphics and Vision, Technical University Graz, Austria), and compared the rate of Nd:YAG laser capsulotomy. The results revealed that the PCO incidence scores were significantly lower for the XY1-implanted eyes compared to AcrySof SN60WF-implanted eyes, and the rates of Nd:YAG laser capsulotomy was 11.4 % in the XY1-implanted eyes and 18.4 % in AcrySof-implanted eyes, with no significant difference. Auffarth et al. [14] analyzed PCO in patients implanted with a XY1 and AcrySof SN60WF using the PCO analysis system, Evaluation of posterior capsule opacification (EPCO) demonstrated that the PCO score of the XY1 implanted eye was significantly lower than that of the eye implanted with the AcrySof SN60WF. Kitaguchi-Iwakiri et al. [15] implanted an AcrySof SN60WF, XY1, and iSert 251/255 (HOYA Surgical Optics) with the same optic shape as the XY1 but without any surface modifications and different haptic materials in patients with a history of noninfectious uveitis. The results revealed that AcrySof had the lowest rate of Nd:YAG laser capsulotomy at 5 years postoperatively, highlighting the effectiveness of plasma surface treatment of Acrysof in suppressing PCO in patients with uveitis [15].

**Table 2:** Summary of XY1 studies.

Author (year)	Patients (eyes)	Follow-up time	Study design	Target IOLs	PCO analysis	PCO score	Rate of Nd:YAG laser capsulotomy
Leydolt et al. [13] (2020)	80 (160)	3 years	Randomized, prospective, patient- and examiner-masked clinical trial with intraindividual comparison	XY1 AcrySof SN60WF	AQUA	XY1: 0.9 ± 0.8 AcrySof SN60WF : 1.4 ± 1.1 (P < 00.001).	XY1: 11.4 % AcrySof SN60WF: 18.6 % (P = 00.23)
Kitaguchi-Iwakiri et al. [15], (2022)	146 (211)	5 years	Retrospective study	AcrySof SN60WF XY1 i Sert 251/255	Slit-lamp grading and fundus visualization	AcrySof SN60WF: 16.2 % XY1: 28.6 % i Sert 251/255: 48.3 % (P < 0.001)	AcrySof SN60WF: 6.3 % XY1: 21.4 % i Sert 251/255: 23.3 % (P < 0.001)
Auffarth et al. [14] (2023)	67 (134)	3 years	Prospective, multicentric, randomized, bilateral, comparative, paired-eye, open-label study	XY1 AcrySof SN60WF	EPCO	XY1: 0.121 ± 0.193 AcrySof SN60WF: 0.239 ± 0.463 (P = 0.026)	XY1: 0 % AcrySof SN60WF: 1.5 %

AQUA: Automated Quantification of After-Cataract; EPCO: evaluation of posterior capsule opacification IOL: intraocular lens; Nd:YAG: neodymium–yttrium–aluminum–garnet; PCO: posterior capsule opacification.

In this study, we quantified PCO at 5 years postoperatively, which is a longer period than those reported in previous studies. Additionally, the cumulative survival of Nd:YAG laser capsulotomy over 10 years for the XY1 implanted in patients without ocular complications was investigated and a notable PCO inhibition effect was revealed. However, the PCO inhibition effect in patients with ocular complications needs to be verified in future studies.

This study had certain limitations. First, among the 30 patients who participated in this study, 25 had interrupted hospital visits and only 5 were followed up for 10 years; therefore, it was impossible to investigate a large number of patients. This loss of follow-up information may be due to most patients with cataracts being older with problematic general conditions that make it difficult for them to continue their hospital visits. Second, the XY1 and FY-60AD had different shapes, which might have affected PCO occurrence. Various factors related to the shape of the IOL, such as the haptics and edge design, are known to impact PCO progression [28]. IOLs with similar shape as the XY1 should be compared to prove the PCO inhibition effect of the XY1's surface modification; however, such IOLs are not currently in clinical use. Third, the EAS-1000 used for PCO analysis failed in 2015 and could no longer be repaired, making analyses impossible after the sixth year. The EAS-1000 is no longer manufactured and sold, thus, we will have to consider new methods of PCO analysis with new equipment. Fourth, the PCO was compared in the right and left eyes of the same patient, although the difference in axial length was not verified. Hecht et al. [29] reported that PCO is more likely to progress in eyes with long axial length because of the insertion of lower powered and thinner optic IOLs, which weaken posterior capsular adhesions and capsular bending. The PCO inhibition effect of the XY1 due to differences in axial length needs to be further investigated. Fifth, because several physicians made decisions regarding the indication for Nd:YAG laser capsulotomy, the timing of capsulotomy may have been affected. Finally, while this study examined the effect of XY1 on PCO inhibition at 10 years postoperatively, further studies investigating the long-term effect of XY1 on PCO inhibition are warranted. This is especially important because the age range for IOL implantation broadens and society continues to age, necessitating the maintenance of postoperative visual function over an extended period.

The results of this study provide clinical evidence for long-term inhibition of PCO in IOLs with surface modifications that improved adhesion between the IOL and posterior capsules. PCO inhibitory effects on surface modified IOLs and IOL surface adhesion continue to attract attention. Recently, surface modified IOLs using new methods such as photodynamic coating, have been investigated [30]. These studies have reported that the hydrophobicity of acrylic IOLs decreases at body temperature, resulting in stronger adhesion between the IOL and the posterior capsule [31]. In order to elucidate a method to completely prevent PCO, it is necessary to verify the PCO suppression effect of XY1 in detail, including comparisons with other surface treatment methods and conduct further research.

## Ethics statement

This study was reviewed and approved by the Institutional Review Board of Dokkyo Medical University, with the approval number: R-69-5J. All patients (or their proxies/legal guardians) provided informed consent to participate in the study. All patients (or their proxies/legal guardians) provided informed consent for the publication of their anonymized case details and images.

## Data availability statement

The data that support the findings of this study are available from the corresponding author, upon reasonable request.

## Funding/support

This study was funded by HOYA surgical optics, Tokyo, Japan. HOYA surgical optics was not involved in the analysis or writing of the manuscript related to this study.

## CRediT authorship contribution statement

**Mayumi Nagata:** Writing – original draft, Resources, Methodology, Investigation, Formal analysis, Data curation, Conceptualization. **Hiroyuki Matsushima:** Formal analysis, Conceptualization. **Tadashi Senoo:** Resources.

## Declaration of competing interest

The authors declare the following financial interests/personal relationships which may be considered as potential competing interestsHiroyuki Mastsushima has received the patent royalty. None of the other authors has any conflicts of interest to disclose.
